# Inflectional and derivational morphological spelling abilities of children with Specific Language Impairment

**DOI:** 10.3389/fpsyg.2014.00948

**Published:** 2014-08-27

**Authors:** Sarah Critten, Vincent Connelly, Julie E. Dockrell, Kirsty Walter

**Affiliations:** ^1^Department of Psychology, Coventry UniversityCoventry, UK; ^2^Psychology, Oxford Brookes UniversityOxford, UK; ^3^Department of Psychology and Human Development, Institute of Education, University of LondonLondon, UK

**Keywords:** spelling, SLI, morphemes, inflectional, derivational, reading, language, writing

## Abstract

Children with Specific Language Impairment (SLI) are known to have difficulties with spelling but the factors that underpin these difficulties, are a matter of debate. The present study investigated the impact of oral language and literacy on the bound morpheme spelling abilities of children with SLI. Thirty-three children with SLI (9–10 years) and two control groups, one matched for chronological age (CA) and one for language and spelling age (LA) (aged 6–8 years) were given dictated spelling tasks of 24 words containing inflectional morphemes and 18 words containing derivational morphemes. There were no significant differences between the SLI group and their LA matches in accuracy or error patterns for inflectional morphemes. By contrast when spelling derivational morphemes the SLI group was less accurate and made proportionately more omissions and phonologically implausible errors than both control groups. Spelling accuracy was associated with phonological awareness and reading; reading performance significantly predicted the ability to spell both inflectional and derivational morphemes. The particular difficulties experienced by the children with SLI for derivational morphemes are considered in relation to reading and oral language.

## 1. Introduction

Children with specific language impairment (SLI) experience problems with the acquisition and processing of oral language. They often have difficulties with the semantic, syntactic and phonological aspects of language and there is some debate around how children with SLI process morphemic affixes. In particular, there is some evidence that children with SLI have more difficulty with inflections suffixes (Montgomery and Leonard, 1998; Marshall and Van Der Lely, 2007; Oetting and Hadley, 2009).

Children with SLI also often have associated literacy difficulties in reading (Botting et al., 2006) and the production of written text (Dockrell et al., 2007). However, the specific difficulties that the children experience with spelling and the cognitive processes responsible for these difficulties remain a matter of debate (Silliman et al., 2006). The current study compares the performance of children with SLI and matched peers in spelling inflectional and derivational morphemes. The extent to which language or literacy skills underpin spelling performance of these different types of affixes is examined.

The ability to coordinate phonemes, orthographic features of written words and the morphological analysis of both base words and bound morphemes underlies the development of spelling (Nagy et al., 2006). Developing an accurate orthographic lexicon to support conventional spelling is an extended process and all three word forms (phonological, orthographic, morphological) are involved from the initial stages of learning to spell (Bahr et al., 2012).

Inflectional and derivational affixes are bound morphemes which play an important role when constructing meaningful text. Inflectional morphemes are suffixes which provide grammatical information about the base words they are bound to through marking, for example, agreement or tense. By contrast derivational morphemes may occur at the beginning (prefixes) or end of a word (suffixes) and produce semantic changes by transforming the grammatical form of a word. Any difficulty in spelling these bound morphemes will impact on the grammatical and semantic accuracy and the complexity of texts produced, and may help partly explain the writing difficulties of children with SLI (Dockrell and Connelly, 2013).

The difficulties experienced by children with SLI in spelling are well established and analysis of single word errors has typically shown a disproportionately high level of phonological spelling errors in the children's texts (e.g., Bishop and Clarkson, 2003). However, to date, the majority of studies have suggested that the spelling performance of children with SLI is commensurate with younger spelling or language matched peers for general spelling ability (Mackie and Dockrell, 2004; Cordewener et al., 2012). In particular, it has been shown that children with SLI consistently spell the root morphemes of inflected and derived words consistently and that this is closely tied and predicted by their general spelling ability and showed no difference when compared to a spelling matched typically developing group of children (Deacon et al., 2014). Therefore, while knowledge of the spelling of a word root can be helpful when dealing with the spelling of inflected or derived forms (Goodwin et al., 2013) the difficulties of children with SLI are thought to lie more with particular aspects of morphology such as suffixes rather than the use of morphemes in general (Oetting and Hadley, 2009).

A number of studies that have focused on the spelling of inflectional morphemes have suggested that children with SLI have a particular weakness with producing regular past tense verbs ending in -*ed* and regular plural nouns ending in -*s* (Windsor et al., 2000; Mackie and Dockrell, 2004; Silliman et al., 2006; Larkin et al., 2013) and often omit them entirely. Two results are of particular importance from the Larkin et al. study (2013). Firstly there were trends that quantitative differences in accuracy might exist not only between the children with SLI and their chronological age matches, but also their younger spelling matches as well. However, the sample was too small to detect significant differences (*N* = 15). Secondly the error patterns differed within the group with SLI, suggesting that participants with weaker phonological skills experienced particular difficulties. Differential spelling difficulties in children with SLI may, thus, reflect the consequence of different vulnerabilities within the language system.

In contrast, the examination of the spelling of derivational morphemes has been comparatively neglected. Silliman et al. (2006) found trends suggesting that children with SLI may be poorer when spelling words comprising phonological and orthographic shifts from the base to the derived form compared to spelling matches again, reporting omission errors. However, the identification of qualitative differences may be obscured by the small sample size (*N* = 8). Given the importance of morphological skills in reading and writing (Green et al., 2003) and the efficacy of morphological instruction, especially for struggling writers (McCutchen et al., 2014) there is a need to further examine the difficulties that children with SLI experience with the spelling of derivational morphemes. Therefore, the first aim of the present study was to examine accuracy and error type when spelling inflectional and derivational morphemes for children with SLI compared to age and language/spelling matches.

The extent to which patterns of performance in spelling morphemes are related to performance on language and literacy measures, is also considered. It is theorized that language abilities should influence spelling via semantic-orthographic connections (Nation and Snowling, 1998a) and oral language skills are associated with spelling ability in typically developing children (e.g., Ouellette and Sénéchal, 2008). However, there have been varied results in determining which language skills may be linked to spelling in children with SLI as neither vocabulary (Dockrell and Connelly, 2013), nor narrative comprehension (McCarthy et al., 2012) significantly predicted spelling ability.

The consistent relationship found between phonological skills and spelling is relatively uncontested. As such it is an important skill to consider when examining links between oral language and spelling in children who are reported to have poor phonological skills (e.g., Bishop and Snowling, 2004; Fraser et al., 2010). Difficulties with phonologically based tasks have been associated with spelling problems in children with SLI (e.g., Bishop and Clarkson, 2003). Both rhyme and nonword reading have been found to predict general spelling ability (Dockrell and Connelly, 2013) and inflectional morpheme spelling (Larkin et al., 2013) respectively, in children with SLI.

However, English is a morphophonemic orthography and therefore spelling also involves an understanding and awareness of the linguistic relationship between sound and meaning. Morphological awareness (particularly of affixes) develops as children learn to recognize the regularities of bound morphemes across many words and has been consistently shown to make a unique contribution to spelling development in typically developing children (e.g., Nagy et al., 2006). Furthermore, inflectional and derivational morphological awareness may contribute to inflectional and derivational morpheme spelling respectively (Apel et al., 2012). To date the relationship between morphological awareness and spelling has not been examined in children with SLI. Children with SLI have poorer levels of morphological awareness in comparison to their same aged peers (Smith-Lock, 1995) and tend to omit inflected forms when speaking (Montgomery and Leonard, 1998; Marshall and Van Der Lely, 2007) and it is predicted that these oral language difficulties with morphology will impact on their spelling performance. Therefore, the second aim of the present study was to examine oral language ability, phonological awareness and morphological awareness in relation to both inflectional and derivational morpheme spelling in children with SLI. Building on the work of Larkin et al. (2013) the current study will examine the relationships between oral language and bound morpheme spelling specifically. The present study will also extend the Larkin et al. (2013) study by considering both derivational and inflectional morphemes.

Although the difficulties experienced with language by children with SLI may lead to consequent spelling problems there is also a close developmental relationship between reading and spelling (e.g., Zutell and Rasinski, 1989; Swanson et al., 2003) and children with SLI struggle with learning to read (Botting et al., 2006). It is, therefore, possible that reading may be an important moderator of spelling in children with SLI. Indeed recent studies have suggested that it is reading skills not oral language that predicts spelling in children with SLI (e.g., McCarthy et al., 2012; Mackie et al., 2013). However, it was general spelling ability that was examined in these studies rather than bound morpheme spelling specifically and the failure to examine specific spelling skills may have masked the influence of specific dimensions of the oral language system.

Error analyses can be used to highlight transitions in the relationship between phonological and morphological knowledge when learning to spell (Nunes et al., 1997; Critten et al., 2007). For example, initially when spelling complex words such as “*filled*” young children may omit the inflectional morpheme, e.g., “*fil*” as initial sounds are the first to be noted while awareness of the final sounds and middle sounds of words develops later (Ehri, 2005). When there is some awareness of the final sounds a phonologically implausible letter string may be supplied for the morpheme using incorrect phoneme-grapheme correspondences, e.g., *filt* where *-t* represents *-ed*. However, once more advanced phonological knowledge starts to develop then children may over-apply phoneme-grapheme correspondences to spell all aspects of a word including the morpheme, e.g., *fild* where *-d* represents *-ed*. It is only when children make correspondences between morphological units in the oral language and their specific spellings and realize that not all words are spelled as they sound that correct application of units such as *-ed* can be observed, e.g., *filed*. Therefore, error analyses can highlight the underlying role of different aspects of oral language in bound morpheme spelling and suggest developmentally how children are progressing in their understanding of bound morphemes in the orthography. Error analysis will be used to investigate qualitative differences in spelling performance.

In this present study, children with SLI were compared to two control groups; one matched for chronological age (CA group) and one younger group matched for language and spelling (LA group). Since children with SLI show consistent spelling of word roots tied to general spelling ability (Deacon et al., 2014) then a spelling matched group should allow us to control for this factor (Goodwin et al., 2013) while concentrating on the suffix issue that the literature points out as a particular difficulty for children with SLI. The three groups were given dictated spelling tasks containing bound morphemes; inflectional morphemes of regular past tense verbs and regular plural nouns and derivational morphemes with phonological and orthographic shifts as indicated by previous findings (e.g., Silliman et al., 2006). An error-coding scheme was employed to focus on the bound morpheme spelling errors in reference to a typical developmental sequence of spelling errors (Critten et al., 2007) and the scheme used by Larkin et al. (2013) for children with SLI.

Finally a detailed assessment of language and literacy skills was conducted. Given the absence of any predictive effects derived for spelling from receptive vocabulary (Dockrell and Connelly, 2013) or narrative comprehension (McCarthy et al., 2012) oral language was measured by an expressive task of sentence generation. Phonological awareness was measured by both rhyme (given the findings of Dockrell and Connelly, 2013) and elision abilities. Morphological awareness was measured in relation to both inflectional and derivational awareness to build on the findings with typical children (Apel et al., 2012). Word reading was also examined (McCarthy et al., 2012; Mackie et al., 2013).

Our first objective was to examine both the accuracy and any errors in the children's spelling of inflectional and derivational morphemes in order to establish any differences between the children with SLI and their matched peers. We predicted that the children with SLI would perform significantly lower than the CA matches but commensurate with the LA matches. By contrast we reasoned, given the indicative data from Larkin et al. (2013) and Silliman et al. (2006) that accurate spelling of inflectional and derivational morphemes for the children with SLI would be poorer than both CA and LA matches and more omission errors would be made. The second objective was to examine which, if any, of our oral language dimensions were associated with inflectional and derivational morpheme spelling. We predicted that both oral morphological and phonological awareness would account for significant amounts of variance but that these associations would be moderated by reading ability.

## 2. Methods

### 2.1. Participants

Ninety-nine children in three matched groups: (a) 33 children identified with SLI; (22 = males, 11 = females), mean age = 9:10 years, *SD* = 3.57 months (range = 11 months). Children of this age were chosen as the spelling of younger children with SLI may be difficult to interpret due to floor effects when required to carry out a complex spelling task involving morphology. (b) 33 children matched for chronological age (CA) and gender, mean age = 9:10 years, *SD* = 2.94 months (range = 10 months) and (c) 33 children matched for gender, language (formulated sentences) and single word spelling abilities (LA), mean age = 8;1 years, *SD* = 6.25 months (range = 7 months). All children had English as their first language and were predominantly of white, British ethnicity. The level of Social Economic Status (SES) was controlled for across schools by checking that the percentage of children receiving free schools meals (a strong indicator of SES in the UK) was in the average range.

To recruit the SLI sample, children were identified across five counties in southern England. Professionals were asked to nominate children who had specific language impairments who participated in a screening process using the four core sub-tests of the Clinical Evaluation of Language Fundamentals, 4th edition (CELF-4 UK, Semel et al., 2006): concepts and following directions, recalling sentences, formulated sentences, word classes receptive and expressive. For a diagnosis of SLI, children had to achieve a standard score of 75 or below (2 SDs below the mean). The matrices test from the British Ability Scales, 2nd Edition (BAS II: Elliott et al., 1997) established non-verbal abilities within the average range. As Table 1 shows all participants met the criteria for SLI, with a significant difference between their CELF-4 test score and their BAS II matrices test: *t*_(64)_ = 15.39, *p* < 0.001, *r* = 0.89. Additional measures examined phonological awareness, morphological awareness and reading and are detailed in Table 2.

**Table 1:** **Means, (standard deviations), f score, df, *p*-value, effect size and Bonferroni *post-hoc* results (where applicable) for screening measures per group: SLI, CA, LA**.

**Measure**	***SLI***	***CA***	***LA***	***F***	***df***	***p***	**Partial η^2^**	**Bonferroni *post-hoc***
Core language standard score (CELF)	68.4 (5.5)	102.9 (11.3)	93.6 (7.9)					
Non-verbal abilities: matrices ability score (BAS)	96.1 (6.5)	104.9 (9.7)	98.9 (7.8)					
**MATCHING VARIABLES**								
Age in years/months (SD in months)	9/10 (3.6)	9/10 (2.9)	8/1 (6.2)	244.3	2,96	<0.001	0.84	SLI = CA > LA
Formulated sentences raw score (CELF)	31.4 (4.2)	47.5 (4.4)	31.2 (4.2)	155.8	2,96	<0.001	0.77	SLI = LA, SLI < CA LA < CA
Spelling raw score (BAS)	16.3 (4.3)	22.3 (5.2)	16.6 (4.8)	16.1	2,96	<0.001	0.25	SLI = LA, SLI < CA LA < CA
Spelling ability score (BAS)	85.0 (16.5)	121.1 (16.3)	94.4 (13)	48.5	2,96	<0.001	0.50	SLI < LA < CA

**Table 2:** **Means, standard deviations, f score, df, *p*-value, effect size and Bonferroni *post-hoc* results for language and literacy measures per group: SLI, CA, LA**.

**Measure**	***SLI***	***CA***	***LA***	***F***	***df***	***P***	**Partial η^2^**	**Bonferroni *post-hoc***
Inflectional morphological awareness raw score	10.9 (1.4)	12.8 (0.36)	12.1 (0.97)	30.4	2,96	<0.001	0.39	SLI < LA < CA
Inflectional morphological awareness z score	−0.79 (1.1)	0.71 (0.29)	0.09 (0.76)	30.4	2,96	<0.001	0.39	SLI < LA < CA
Derivational morphological awareness raw score	5.1 (1.1)	5.8 (0.48)	5.3 (0.95)	4.9	2,96	0.009	0.09	SLI < CA SLI = LA CA = LA
Derivational morphological awareness z score	−0.3 (1.2)	0.41 (0.52)	−0.11 (1.0)	4.9	2,96	0.009	0.09	SLI < CA SLI = LA CA = LA
Phonological elision raw score (CTOPP)	10.8 (4.3)	17.2 (2.9)	14.2 (4.2)	22.9	2,96	<0.001	0.32	SLI < LA < CA
Phonological rhyme raw score (PhAB)	12.3 (4.5)	18.4 (3.1)	17.1 (3.4)	24.6	2,96	<0.001	0.34	SLI < CA SLI < LA CA = LA
Phonological awareness z score (elision z + rhyme z)	−1.5 (1.58)	1.2 (1.0)	0.29 (1.3)	36.5	2,96	<0.001	0.43	SLI < LA < CA
Single word reading raw score (YARC)	31.6 (11.0)	49.2 (5.8)	38.7 (8.0)	35.0	2,96	<0.001	0.42	SLI < LA < CA
Single word reading z score (YARC)	−0.73 (0.9)	0.84 (0.52)	−0.09 (0.72)	35.0	2,96	<0.001	0.42	SLI < LA < CA

The two groups of comparison children attended the same primary schools as those diagnosed with SLI, and were selected by teachers on the basis of average attainment on curriculum assessments and no additional learning needs. The CA comparison children were confirmed as having language ability and non-verbal ability within the normal range using the same CELF-4 UK core tests and the BAS II matrices and were matched in age to the children with SLI within 3 months and did not differ overall in age.

The LA comparison children also had scores on language and non-verbal ability within the average range and were matched with the children with SLI using their raw score on the formulated sentences task from the CELF-4 UK. The LA comparison children were also matched to the SLI group using their raw score on the single word spelling task from the BAS II. Despite the fact that the CA group was chosen purely for their age they scored significantly higher than the other two groups for non-verbal ability although the SLI and LA groups did not differ.

### 2.2. Measures

#### 2.2.1. General language ability

Clinical Evaluation of Language Fundamentals (CELF-4 UK, Semel et al., 2006). The CELF provides core sub-tests of receptive and expressive language abilities. This produces a Total Language Score that can be utilized for the identification of language impairment. Children from the SLI and CA groups were screened for language ability using the four core sub-tests for 9–16 years: (a) *Concepts and following directions*; children are shown pictures and asked to identify items and/or point to them in a prescribed order according to a verbal instruction, (b) *Recalling sentences*; children are asked to imitate orally presented sentences, (c) *Formulated sentences*; children are shown a picture of a scene and asked to verbalize a sentence that both describes the picture and includes a target word, (d) *Word classes*; children are verbally presented with four words and asked to first identify the two words that go together (receptive component) and then to explain why they go together (expressive component). The children from the LA group were given the four core sub-tests for 5–8 years where the *word classes* task is replaced by *word structure*; children are shown pictures and asked to describe them using a verbal prompt designed to elucidate understanding of word class and morphology. Reliability for the core sub-tests for 9–10 years, 0.94 and for 5–8 years, 0.95–0.96.

#### 2.2.2. Non-verbal ability

The British Ability Scale II (BAS II) Matrices subtest (Elliott et al., 1997). Children are presented with a set of patterns presented in a four or six part grid where one part of the grid is incomplete and children are required to select the missing piece from six possible responses; reliability 0.85, validity with the WISC-III performance scale 0.47

#### 2.2.3. Spelling

The British Ability Scale II (BAS II) Spelling subtest (Elliott et al., 1997). Children are verbally presented with a series of phonetically regular and irregular monosyllabic and bisyllabic words. The words are first presented in isolation, then within the context of a sentence and finally in isolation and asked to respond by writing the word: reliability 0.91.

#### 2.2.4. Phonological awareness

Complete Test Of Phonological Processing (CTOPP; Wagner et al., 1999) and Phonological Assessment Battery (PhAB; Frederickson et al., 1997). (1) Children were tested on the elision task from the CTOPP which requires identification and segmentation of the different phonological units within words, reliability, 0.80; validity with the Woodcock Reading Mastery Test—R (Word Attack and Word Identification sub-tests) 0.49–0.84 and (2) A test of rhyme from the PhAB where children chose two words that rhyme out of a choice of three (one irrelevant word and two that rhyme); reliability ≥0.80; validity with the Neale Analysis of Reading Ability (NARA; Neale et al., 1997) reading accuracy 0.24–0.56.

#### 2.2.5. Inflectional and derivational morphological awareness

A test of morphological awareness was created from selected items on the CELF-4 UK (Semel et al., 2006): only items assessing awareness of inflectional morphemes (*N* = 13) and derivational morphemes (*N* = 6) were used in the current study. An example of an inflectional item is to show children a picture of a horse and say “Here is one horse,” then another picture with two horses is pointed to: “Here are two …” and the child has to supply the word with the correct inflected morpheme of *-s*. An example of a derivational item is to show a picture of a teacher and say: “This man teaches. He is called a …” and the child has to supply the word with the correct derived morpheme of *-er.*

#### 2.2.6. Reading

York Assessment of Reading Comprehension (YARC) Passage reading (Snowling et al., 2009). Children were given the Single Word Reading Task (SWRT) comprising 60 words presented on a card and asked to read them aloud, reliability, 0.85.

#### 2.2.7. Experimental morphological spelling tasks

A list of 42 words was developed and presented as two 21 word spelling tests, delivered in a randomized order. The majority of words were derived from previous studies conducted with children aged 7–11 years and so were considered appropriate for the ages of the sample in this study. Written word frequency analyses had been completed by the original researchers for inflectional words ending in -*ed* (Nunes et al., 1997) and -*s* (Kemp and Bryant, 2003) and derivational words including phonological, orthographic and phonological and orthographic shifts (Mossing et al., 2009; Wiggins et al., 2010) to establish comparable levels within the morpheme types. Furthermore, for the present study written word frequency was also checked using the UK derived Children's Printed Word Database (Masterson et al., 2003). This demonstrated that the frequency of the inflectional words ranged from 3 to 1652 and that the derivational words were generally less frequent, as would be expected, ranging from 3 to 533. See in supplemental materials for the complete word list and written word frequency scores.

#### 2.2.8. Inflectional morphemes

Derived and adapted from Nunes et al. (1997) and Kemp and Bryant (2003). There were 24 words containing inflectional morphemes; 12 regular past tense verbs containing *-ed*, e.g., *filled* and 12 regular plural nouns, e.g., *trees*.

#### 2.2.9. Derivational morphemes

Derived and adapted from Silliman et al. (2006), Mossing et al. (2009) and Wiggins et al. (2010). There were 18 words containing derivational morphemes: six where there was a phonological shift from the base word to the derived form, e.g., *different*, six where there was an orthographic shift from the base word to the derived form, e.g., *attention* and six where there were both phonological and orthographic shifts, e.g., *student*.

### 2.3. Procedure

All children were assessed individually in a quiet room at school. Ethical approval for the study had been gained in line with guidelines from the British Psychological Society (BPS) through the university ethics committee and informed consent from schools, parents and children was provided prior to any testing. During the screening process the CELF core tests, BAS matrices and BAS spelling were administered in two testing sessions. The two morphological spelling tasks and the phonological awareness and morphological awareness tasks were delivered over two further testing sessions. Children were allowed to terminate the sessions if they wished. However, no child terminated the sessions since the organization of data collection into different sessions resulted in manageable time periods of testing for the children.

All standardized tests were administered according to the procedures in the manual. For the morphological spelling tasks, each word was verbally presented in isolation, in the context of a sentence and then in isolation again and children were asked to write out the word.

### 2.4. Classification of spelling errors within the morphological spelling tasks

The focus was only on the spelling of the inflectional or derivational morpheme within each word, i.e., the spelling of the base was not analyzed further. Morphemes which were incorrectly spelled were categorized into one of the following mutually exclusive error types (Larkin et al., 2013) (1) Omission where the morpheme was not attempted at all, e.g., *fill* as an error attempt of *filled*, or *atten* as an error attempt of *attention* (2) Phonologically implausible where the morpheme was attempted (incorrectly) but the phoneme-grapheme correspondences did not produce a correct pronunciation, e.g., *fillt* where *-t* is not a phonologically plausible version of *-ed* or *attensed* where *-sed* is not a phonologically plausible version of *-tion*, (3) Phonologically plausible where the morpheme was again incorrectly spelled but the phoneme-grapheme correspondences did produce a correct pronunciation of the target morpheme, e.g., *filld*, where *-d* is a phonologically plausible attempt for *-ed*, or *attenshun* where -*shun* is a phonologically plausible attempt for *-tion*. The spelling errors were coded by two of authors of this paper and achieved an inter-rater reliability of 100%.

## 3. Results

The results are presented in three sections. Section 1 examines group differences in children's spelling performance according to morpheme type. Section 2 examines associations between to inflectional and derivational morphological spelling abilities and language and literacy measures. Finally Section 3 examines predictors of children's inflectional and derivational morphological spelling ability using hierarchical regressions.

### 3.1. Group differences in morphological spelling ability

Means (SD) of group performance for each morpheme type are presented in Table 3. A Mixed ANOVA with group (between subjects factor) and morpheme type; inflectional and derivational (within subjects factor) was conducted for the number of words (base + morpheme) spelled correctly. Non-verbal ability and chronological age were added as co-variates although neither were significant [non-verbal ability *F*_(1, 94)_ = 0.09, *p* = ns; chronological age *F*_(1, 94)_ = 0.01, *p* = *ns*]. There was a main effect of group *F*_(2, 94)_ = 30.62, *p* < 0.001, η *p*^2^ = 0.39 and morpheme type *F*_(2, 94)_ = 9.69, *p* = 0.002, η *p*^2^ = 0.09 confirming that more words containing inflectional morphemes were correctly spelled and also a significant interaction between group and morpheme type *F*_(2, 94)_ = 8.17, *p* = 0.001, η *p*^2^ = 0.15. Subsequent multivariate ANOVAs confirmed that there were group differences for both the number of words containing inflectional morphemes correctly spelled *F*_(2, 94)_ = 31.21, *p* < 0.001, η *p*^2^ = 0.39 and the number of words containing derivational morphemes correctly spelled *F*_(2, 94)_ = 34.26, *p* < 0.001, η *p*^2^ = 0.42. Bonferroni *post-hoc* analyses revealed that for both word types, the SLI and LA groups did not differ but were significantly less accurate than the CA group (*p* < 0.001).

**Table 3:** **Means and standard deviations for the number of words and morphemes spelled correctly and number and proportions of error types (omission, phonologically implausible, non-phonologically plausible) according to Group (SLI, CA, LA) and morpheme type (Inflectional, Derivational)**.

**Morpheme type**	***SLI***	***CA***	***LA***
**INFLECTIONAL (/24)**			
Correct words	9.2 (5.9)	18.6 (3.7)	11.4 (5.3)
Correct morphemes	16.8 (5.0)	22.0 (3.0)	17.9 (3.9)
Omission: number	1.6 (2.3)	0.1 (0.4)	0.5 (0.9)
Omission: proportion	0.2 (0.2)	0.2 (0.4)	0.1 (0.1)
Phonologically implausible: number	0.3 (0.6)	0.0 (0.1)	0.1 (0.6)
Phonologically implausible: proportion	0.0 (0.1)	0.0 (0.0)	0.0 (0.0)
Phonologically plausible: number	5.4 (4.2)	1.3 (2.2)	5.1 (3.3)
Phonologically plausible: proportion	0.8 (0.2)	0.8 (0.3)	0.9 (0.2)
**DERIVATIONAL (/18)**			
Correct words	3.1 (3.0)	9.8 (4.2)	4.3 (3.1)
Correct morphemes	5.9 (3.9)	12.9 (2.9)	8.5 (4.0)
Omission: number	1.1 (1.8)	0.0 (0.0)	0.2 (0.8)
Omission: proportion	0.1 (0.1)	0.0 (0.0)	0.0 (0.0)
Phonologically implausible: number	5.5 (3.4)	0.7 (1.2)	3.0 (3.39)
Phonologically implausible: proportion	0.4 (0.2)	0.1 (.1)	0.3 (0.2)
Phonologically plausible: number	5.4 (2.4)	4.0 (2.1)	6.4 (3.0)
Phonologically plausible: proportion	0.5 (0.2)	0.9 (0.1)	0.7 (0.3)

This analysis was then repeated using the same factors and co-variates but this time with the accuracy scores for the spelling of the morphemes alone, again neither co-variate was found to be significant; non-verbal ability *F*_(1, 94)_ = 1.85, *p* = ns and chronological age *F*_(1, 94)_ = 0.08, *p* = ns. As before there was a main effect of group *F*_(2, 94)_ = 27.33, *p* < 0.001, η *p*^2^ = 0.37 and morpheme type *F*_(1, 94)_ = 19.21, *p* < 0.001, η *p*^2^ = 0.17 and a significant interaction between group and morpheme type *F*_(2, 94)_ = 6.43, *p* < 0.001, η *p*^2^ = 0.12. Subsequent multivariate ANOVAs confirmed that there were group differences for both the number of inflectional morphemes correctly spelled *F*_(2, 96)_ = 14.98, *p* < 0.001, η *p*^2^ = 0.24 and the number of derivational morphemes correctly spelled *F*_(2, 96)_ = 31.78, *p* < 0.001, η *p*^2^ = 0.40. In both cases the effect sizes were large. Bonferroni *post-hoc* analyses revealed that for the inflectional morphemes the SLI and LA groups did not differ but were significantly less accurate than the CA group (*p* < 0.001). In contrast the three groups differed in their performance on derivational morphemes where the SLI group was less accurate than both the CA (*p* < 0.001), and LA (*p* < 0.001), groups and the LA group was poorer than the CA group (*p* < 0.001).

To examine this further the proportions of error type were compared between the groups (between subjects factor). Table 3 presents the number and proportions of the types of errors made by the three different groups. Some children (CA group *N* = 19, SLI group *N* = 2) made no inflectional morpheme spelling errors and were therefore excluded from the analysis. Overall all three groups tended to make phonologically plausible errors when spelling inflectional morphemes and the number of omissions and phonologically implausible attempts were negligible. Thus, there were no group differences for omission errors: *F*_(2, 75)_ = 2.39, *p* = ns, phonologically implausible errors: *F*_(2, 75)_ = 0.72, *p* = ns or phonologically plausible errors: *F*_(2, 75)_ = 2.65, *p* = ns.

For derivational morphemes, all children made at least one spelling error and therefore no child was excluded from the analysis. Group differences were apparent when exploring error type for omission errors: *F*_(2, 96)_ = 9.32, *p* < 0.001, η *p*^2^ = 0.16, phonologically implausible errors: *F*_(2, 96)_ = 19.08, *p* < 0.001, η *p*^2^ = 0.28 and phonologically plausible errors: *F*_(2, 96)_ = 19.07, *p* < 0.001, η *p*^2^ = 0.33. The largest effect was evident for phonologically plausible errors whereas the difference for omission errors was negligible. Bonferroni *post-hoc* analyses revealed that for the phonologically implausible errors the SLI group made proportionately more than both the CA (*p* < 0.001), and LA (*p* < 0.001), groups and the LA group made more of both type of error than the CA group (*p* < 0.001). In contrast for the phonologically plausible errors, the SLI made proportionately fewer compared to both the CA (*p* < 0.001), and LA (*p* < 0.001), groups and the LA group made fewer than the CA group (*p* < 0.001).

### 3.2. The relationships between inflectional and derivational morphological spelling, language and reading measures

Group correlations (partialling out non-verbal ability) were conducted for inflectional morpheme spelling ability (number of inflectional morphemes spelled correctly), derivational morpheme spelling ability (number of derivational morphemes spelled correctly), oral language ability (CELF Formulated sentences), phonological ability (combined z scores from the CTOPP elision and PhAB rhyme tasks), inflectional morphological awareness, derivational morphological awareness and word reading ability (YARC single word reading test) and are shown in the supplemental materials link for Table 4. To control for Type I errors, a Bonferroni correction was computed at 6/0.05 = 0.008.

**Table 4:** **Correlations between various measures according to group (SLI, CA, LA) controlling for non-verbal ability**.

**Group**	**2**	**3**	**4**	**5**	**6**	**7**
**SLI**						
1. Inflectional morpheme spelling ability raw score	0.71^**^	0.14	0.62^**^	0.07	0.03	0.84^**^
2. Derivational morpheme spelling ability raw score		0.02	0.61^**^	0.23	0.19	0.83^**^
3. Oral language ability raw score (CELF Formulated sentences)			0.11	0.14	0.16	0.05
4. Phonological ability z score (CTOPP elision + PhAB Rhyme)				0.40^*^	0.02	0.69^**^
5. Inflectional morphological awareness raw score					0.12	0.14
6. Derivational morphological awareness raw score						0.08
7. Word reading raw score (YARC)						_
**CA**						
1. Inflectional morpheme spelling ability raw score	0.66^**^	0.15	0.47^**^	0.21	0.30	0.40^*^
2. Derivational morpheme spelling ability raw score		0.01	0.34	0.33	0.40^*^	0.51^**^
3. Oral language ability raw score (CELF: Formulated sentences)			0.03	0.03	0.13	0.00
4. Phonological ability z score (CTOPP elision + PhAB Rhyme)				0.08	0.42^*^	0.50^**^
5. Inflectional morphological awareness raw score					0.02	0.20
6. Derivational morphological awareness raw score						0.48^**^
7. Word reading raw score (YARC)						_
**LA**						
1. Inflectional morpheme spelling ability raw score	0.46^**^	0.0	0.46^**^	0.07	0.05	0.60^**^
2. Derivational morpheme spelling ability raw score		0.17	0.51^**^	0.03	0.14	0.68^**^
3. Oral language ability raw score (CELF Formulated sentences)			0.37^*^	0.03	0.15	0.02
4. Phonological ability z score (CTOPP elision + PhAB Rhyme)				0.03	0.23	0.65^**^
5. Inflectional morphological awareness raw score					0.03	0.07
6. Derivational morphological awareness raw score						0.03
7. Word reading raw score (YARC)						_

** *Significant at 0.008 (Bonferroni adjusted for multiple comparisons)*.

* *Significant at 0.05*.

For both the SLI and LA groups, there were significant relationships between inflectional and derivational morphological spelling ability and phonological and word reading abilities. However, for the CA group while the relationships with phonological and reading abilities remained for inflectional morphological spelling, for derivational spelling the relationship with phonological awareness was no longer significant but rather derivational morphological spelling ability was significantly related to derivational morphological awareness. Notably there were no significant relationships between oral language ability and morphological spelling in any group while phonological and reading abilities were correlated for all groups. Furthermore, reading related to derivational morphological awareness but only for the CA group and oral language ability related to phonological ability but only for the LA group.

### 3.3. Predictors of inflectional and derivational morphological spelling

Hierarchical regressions (See Table 5) were used to examine the predictors of inflectional and derivational morphological spelling ability. Analyses were collapsed across the groups to provide sufficient power to address this question. The first regression analyses examined predictors of inflectional morphological spelling ability. Non-verbal ability and chronological age was entered in the first step, followed by oral language ability, inflectional morphological awareness, phonological awareness, and word reading in the second step. The model from the first step did not prove significant [*F*_(2, 95)_ = 16, *p* = ns, Adjusted *R*-square = 0.02]. However, once the variables in the second step were added a significant model did emerge [*F*_(6, 91)_ = 26.79, *p* < 0.001, Adjusted *R*-square = 0.62, *r*-square change = 0.64] and demonstrated that non-verbal ability and word reading were the only significant predictors of inflectional morphological spelling ability and that chronological age, oral language ability, inflectional morphological awareness and phonological awareness, did not significantly contribute to explaining the variance.

**Table 5:** **Summary of the final model of hierarchical regressions analysis when predicting inflectional and derivational morphological spelling ability**.

**Variable**	***B***	***SE B***	**Beta**	***T***	***p***
**PREDICTING INFLECTIONAL MORPHOLOGICAL SPELLING**					
Non-verbal ability (BAS matrices)	−0.086	0.032	−0.185	−2.67	0.009
Chronological age	0.031	0.032	0.074	0.95	*ns*
Oral language ability raw score (CELF: Formulated sentences)	−0.009	0.050	−0.017	−0.176	*ns*
Inflectional morphological awareness raw score (CELF: Word Structure)	0.286	0.267	0.079	1.07	*ns*
Phonological ability z score (CTOPP elision + PhAB Rhyme)	0.455	0.284	0.173	1.60	*ns*
Word reading raw score (YARC)	0.267	0.044	0.655	6.13	<0.001
**PREDICTING DERIVATIONAL MORPHOLOGICAL SPELLING**					
Non-verbal ability (BAS matrices)	−0.030	0.031	−0.062	−0.972	*ns*
Chronological age	0.032	0.030	0.074	1.07	*ns*
Oral language ability raw score (CELF: Formulated sentences)	0.034	0.044	0.064	0.779	*ns*
Derivational morphological awareness raw score (CELF: Word Structure)	0.128	0.316	0.025	0.404	*ns*
Phonological ability z score (CTOPP elision + PhAB Rhyme)	0.384	0.268	0.143	1.43	*ns*
Word reading raw score (YARC)	0.283	0.040	0.680	7.04	<0.001

A second regression analyses examined predictors of derivational morphological spelling ability. Variables were entered in the same steps as the inflectional morphology regression although derivational morphological awareness was entered in place of inflectional awareness. The model from the first step did not prove significant [*F*_(2, 95)_ = 1.78, *p* = ns, Adjusted *R*-square = 0.02]. However, once the variables in the second step were added a significant model did emerge [*F*_(6, 91)_ = 34.19, *p* < 0.001, Adjusted *R*-square = 0.67, *r*-square change = 0.66] and demonstrated that word reading was the only significant predictor of derivational morphological spelling ability and that non-verbal ability, chronological age, oral language ability, inflectional morphological awareness and phonological awareness, did not significantly contribute to explaining the variance.

## 4. Discussion

### 4.1. Inflectional morpheme spelling

Previous studies of inflectional morpheme spelling indicated that children with SLI might be poorer at spelling regular past tense and plural morphemes and these inflections would be frequently omitted in comparison with both CA and LA matched peers. However, it was found that the children with SLI were as proficient at spelling inflectional morphemes as their language and spelling ability matched peers but both these groups of children were poorer at spelling inflectional morphemes than their chronological age matched peers. These results demonstrate that performance in spelling ability is more predictive of how accurately these suffixes are spelt rather than morphological awareness. Other studies that have examined both general spelling ability (Cordewener et al., 2012) and the spelling of word roots in children with SLI (Deacon et al., 2014) have reported similar results.

When children failed to spell the inflection accurately the pattern of errors across the groups were broadly similar. There was a predominance of phonologically plausible errors and a very small proportion of phonologically implausible error types. Therefore, most children were employing the developmentally sophisticated strategy of using phoneme-grapheme correspondences (as evidenced by error category) when attempting to spell these morphemes. The minority of omission errors for the SLI group was surprising given previous research (Larkin et al., 2013). However, our larger sample of children with SLI was slightly older than the Larkin et al. (2013) sample and might be showing a benefit of longer experience at school.

All groups showed relationships between inflectional morpheme spelling and phonological awareness. However, no group showed a relationship between inflectional morpheme spelling and either measure of morphological awareness or our measure of expressive oral language. Finally for all groups, there were strong relationships between inflectional morpheme spelling and reading. Thus, it is apparent that in the current cohort inflectional morpheme spelling was associated with the quality of the underlying orthographic and phonological representations that are most often associated with spelling and reading skills. Thus, although the children with SLI seem delayed in their spelling of inflectional morphemes compared to chronological age matches, their spelling ability is underpinned by the same factors as their language and spelling matches. This further confirms previous findings with children with SLI (Dockrell et al., 2007, 2009; Dockrell and Connelly, 2013).

Contrary to other work with typically developing children (e.g., Apel et al., 2012) we found no relationship between inflectional morphological awareness and inflectional spelling in any of the groups sampled despite the fact that inflectional awareness seems quite well developed overall. Therefore, while inflectional morphological awareness could potentially still be contributing to the children's general knowledge of English spelling, phonological and orthographic knowledge are likely forming the representational basis for inflectional morpheme spelling rather than awareness of inflectional morphemes specifically.

### 4.2. Derivational morpheme spelling

The SLI group were less accurate when spelling derivational morphemes compared to both control groups, despite being matched for language and spelling with the LA group, and they also made proportionately more phonologically implausible errors. This study confirms previous research that suggested the SLI group might struggle when spelling words containing phonological and orthographic shifts from the base to derived forms Silliman et al. (2006). Children in the control groups were generally making errors in a phonologically plausible manner. In contrast the SLI group were unable to apply phoneme-grapheme correspondences plausibly when attempting to spell the morpheme, e.g., *-sed for -tion* in *attention* and *-ets* for *-ity* in *majority*.

However, despite these differences in accuracy and error type the SLI group and their LA matches showed similar links between derivational morpheme spelling, phonological awareness and word reading. However, the poorer phonological and reading skills of the SLI group did not allow them to match the performance of the LA group for these more challenging derivations. It could be hypothesized that children with SLI are displaying a difficulty with the semantic links between language and spelling in relation to these derivational morphemes. However, the fact that they achieved parity on the derivational morphological awareness task with the LA group might rule that out. Instead it might be more plausible to suggest that the lower phonological and reading abilities the children with SLI are being more strongly highlighted when the difficulty of the bound morpheme spelling demands increase, showing a specific impairment in the underlying representations of these derivational morphemes.

The older CA group showed a different pattern of relationships whereby successful derivational morpheme spelling was related to derivational morphological awareness and not phonological awareness. They were showing a close link between a complex language task and their spelling ability. The reading skills of the CA group also showed an association with derivational morphological awareness unlike the children with SLI and the LA group so that derivational morphological awareness may be reliant on an appropriate level of reading.

The regressions provided consistent findings. Out of the four key predictors tested, word reading was the only significant predictor when spelling inflectional and derivational morphemes. The predominance of word reading confirms findings from studies of general spelling ability (McCarthy et al., 2012) that it is the strength of underlying orthographic representations rather than dimensions of oral language that may primarily determine spelling attainment. This further demonstrates the close developmental relationship between single word reading and spelling (Zutell and Rasinski, 1989; Swanson et al., 2003).

Inflectional and derivational morphological awareness were not predictive of overall sample performance as had been suggested by some studies of typically developing children (Nagy et al., 2006). However, Nunes and Bryant (2009) argue that explicit understanding and awareness of morphemes may not be crucial for correctly spelling all morphemes and that it can often be achieved by word specific knowledge and in appropriate instances, by the application of phoneme-grapheme correspondences. Therefore, like expressive oral language ability, morphological awareness may have more of an impact later in development. At this point, bound morpheme spelling for the SLI and LA groups is determined by orthographic representations and most likely their connections to phonological awareness rather than morphological awareness. It is also likely that children will revert to phonological strategies if there is any uncertainty when spelling derivational morphemes as these are more challenging for all children in this age range, not just the SLI sample.

### 4.3. Limitations and future directions

Language is a complex skill and the current study used one measure of expressive language to evaluate performance in this area. While there were strong theoretical and empirical reasons to use the expressive language task it could be that this test was not sensitive enough to tap into connections between oral language and morphological spelling. Furthermore, it could be argued that morphological skills also reflect receptive and expressive vocabulary given the hypothesized semantic link to derived morphemes in particular. Thus, consideration of the breadth and depth of children's vocabulary levels at different developmental phases in relation to spelling would further our understanding of the relationships between morphological spelling ability and semantic representations.

Similarly consideration should also be given to the way that inflectional and derivational morphological awareness is measured. We have already outlined the rationale for the tasks that were used, however there is some concern about the possible ceiling effects in both tasks and small effect sizes for the group differences and therefore future tasks could utilize words/bound morphemes that link directly to those included in the spelling tasks. Given the semantic aspect of bound morphemes (derivational morphemes in particular) it would also be interesting to compare spelling of the words in isolation and within sentences to examine contextual influences.

Another possible limitation of this study was the focus on the spelling performance of the bound morphemes specifically, rather than an examination of the ability to spell the root or base word in comparison to the inflected and derived forms. This is particularly pertinent for interpreting our derivational shift word findings as recent work examining typically developing children shows that accuracy when reading derived forms is determined by accuracy when reading the root words (Goodwin et al., 2013). However, other recent research has also shown, as we have, that the spelling of root words by children with SLI are consistent across both root and derived forms and are no worse that spelling matched children (Deacon et al., 2014). Nonetheless, further study examining the frequency of the roots and derived forms and the degree of phonological orthographic and semantic opacity would be very useful.

In conclusion, the current study has demonstrated the importance of reading skills in the spelling performance of typically developing children and those with SLI. Further we have shown that inflectional and derivational spelling may provide a window into the spelling difficulties experienced by pupils with SLI. Further research should examine these conclusions with children at different phases of spelling development, more elaborate measures of morphological awareness and a consideration of the relationship between the root words and the derived forms.

### Conflict of interest statement

The authors declare that the research was conducted in the absence of any commercial or financial relationships that could be construed as a potential conflict of interest.
