# Supplementary material for: Inflectional and derivational morphological spelling abilities of children with Specific Language Impairment
Source: Front Psychol. 2014 Aug 27;5:948. doi: 10.3389/fpsyg.2014.00948 (PMC4145714; doi:10.3389/fpsyg.2014.00948)
Supplement: Supplementary file 1 [file DataSheet1.DOCX]

Appendix 1: *Words used in the two morphological spelling tasks according to inflectional (regular past tense verbs, regular plural nouns) and derivational (phonological shift, orthographic shift, phonological and orthographic shift) morpheme type. Written word frequency (Masterson et al. 2003) is presented in parentheses.*

| Spelling test | Inflectional: Regular past tense verbs | Inflectional: Regular plural nouns | Derivational phonological shift | Derivational orthographic shift | Derivational: phonological and orthographic shift |
| --- | --- | --- | --- | --- | --- |
| One | Called (1652)  Covered (154)  Filled (114)  Laughed (646)  Kissed (16)  Dressed (62) | Birds (441)  Curtains (41)  Tables (22)  Trees (511)  Fleas (38)  Queues (3) | Different (533)  Disappear (24)  Majority (3) | Juicy (22)  Attention (14)  Argument (5) | Student (11)  Excellent (5)  Pleasant (3) |
| Two | Killed (30)  Opened (333)  Started (398)  Stopped (454)  Missed (51)  Learned (41) | Houses (333)  Eyes (498)  Fields (92)  Fees (3)  Drawers (8)  Jaws (35) | Magician (3)  Discussion (3)  Convertible (3) | Hungry (330)  Scary (32)  Easily (54) | Natural (11)  Severity (3)  Confidence (3) |
